# Physiology Vindicated, in a Critique on Liebig’s Animal Chemistry

**Published:** 1843-12

**Authors:** 


### REVIEWS.

---

Art. III.—*Physiology Vindicated, in a Critique on Liebig's Animal Chemistry.* By Charles Caldwell, M.D. Jeffersonville, Ia: A. Spaulding Tilden, 1843. pp. 95, 8vo.

Professor Caldwell is a ready and copious writer. His prolific pen, in the course of a long and industrious life, has been employed in the discussion of most of the leading topics belonging to physiology. It has fallen in our way to have to speak of many of these productions, and generally we have been as forcibly impelled by a sense of justice, as predisposed by our feelings of personal regard for their author, to speak of them in terms of decided approbation. But in the present instance our language must be different, and on that account the task of reviewing the "Critique" is one which we assume with no pleasure. If we could have pronounced it an able paper, though coming short of its aim; if, among its numerous arguments against animal chemistry, one solid objection had been adduced; if the author had even succeeded in pointing out the defects which, undeniably, attach to the doctrines of Liebig, we should have felt pleasure in laying before our readers all that could be urged against those popular theories by one of the most learned physiologists of our country. But after a most careful examination of what Professor Caldwell has written in this work, we cannot admit that he has set forth a single valid reason, why the chemical theories of animal heat and digestion should not be adopted. The argument evinces the learning and research, which we had a right to expect, and it is proposed with an earnestness which shows that the author had a deep conviction of the soundness of his opinions, but it will surprise us if any one, who has studied the writings of Liebig, and is acquainted with the subjects in dispute, deems it so much as plausible. Whatever of force the objections urged might have had in the time of John Hunter, it is not hazardous to say, that by the great body of physiologists they are now regarded as obsolete.

We have admitted the sincerity of Dr. Caldwell in all the opinions advanced in the "Critique," but, in very truth, if we did not know that he is not given to irony, we should strongly suspect him of experimenting upon the credulity of his readers in the following paragraph:

"Convince any man, however high his political and social rank, and his influence among his fellows, that he is nothing but an aggregate of oxygen, nitrogen, carbon, and hydrogen, and a few other lifeless ingredients, put together, fashioned, and held together, by the same affinities, and governed, in the performance of his functions, by the same laws that preside *in* and *over* masses of dead matter—convince *any* man of this, and you necessarily diminish his self-respect, and render him comparatively indifferent to his actions, and regardless of his destiny. Convince mankind at large of this, and you brutify them. Degraded in their own estimation, and approximated in their belief to masses of brute matter, their feelings and conduct will conform to their view of their humbled condition. For, that a sense of high and honorable descent and condition influences morals and actions, as well as manners and bearing, is a maxim as true as any other that belongs to the history and philosophy of man." p. x.

The poet has it,

> "For women are like tricks by sleight of hand,
> Which to admire you must not understand;"

but the learned author of the "Critique" carries the idea much further. He insists that men not only will not "admire" themselves if they get to understand the stuff they are made of, but will be "brutified" by the knowledge. To quote his own words,

"Not only is chemical physiology physically groundless; it is morally pernicious. Chemical physiology and pathology, and chemical practice, the result of them, have slain their millions. From them have arisen the unspeakable mischiefs and miseries of humoralism." p. xi.

We quote these passages, not with any intention of commenting on the sentiments, but to show the spirit and manner in which Professor Caldwell resists the application of chemical philosophy to the functions of animals. No comment can be necessary. The reader has but to turn to any modern book on physiology, for a satisfactory reply to all such objections and denunciations as these. So we proceed, without further preface, to the main argument of the "Critique"—that directed against the chemical theory of *animal heat.* That theory is thus briefly stated by Liebig:

"The mutual action between the elements of the food and the oxygen conveyed by the circulation of the blood to every part of the body, is the source of animal heat." *An. Chem.* p. 17.

"To make use of a familiar, but not, on that account, a less just illustration, the animal body acts, in this respect, as a furnace, which we supply with fuel. In order to keep up, in the furnace, a constant temperature, we must vary the supply of fuel according to the external temperature: that is, according to the supply of the oxygen.

"In the animal body the food is the fuel; with a proper supply of oxygen we obtain the heat given out during its oxidation or combustion." *Ib.* p. 20.

"Those animals which respire frequently, and consequently consume much oxygen, possess a higher temperature than others, which, with a body of equal size to be heated, take into the system less oxygen. The temperature of a child (102°) is higher than that of an adult (99.5°). That of birds (104° to 105.4°) is higher than that of quadrupeds (98.5° to 100.4°), or than that of fishes or amphibia, whose proper temperature is from 2.7° to 3.6° higher than that of the medium in which they live." *Ib.* p. 18.

Such is the theory of animal heat proposed by Liebig, some parts of which he owes to former chemists, but which was never presented in so perfect a shape as it appears in his admirable work. Dr. Caldwell regards it as an entire failure.

We shall examine his reasons for this belief, one of which is stated in the following extract:

"During the hottest period of hot climates, the heat, wasted by the human body, through atmospherical influence, does not amount, on an average, to more than 5°, perhaps not so much. That quantity, therefore, and no more, must be supplied by the calorific process of the system.

"But, during the winters of the frozen north, the heat, abstracted from the body of man, by atmospherical agency, amounts, not unfrequently, to from 140° to 150°. This is from twenty-eight to thirty times as much as is abstracted under the influence of tropical heat. In such a case, therefore, the calorific process must supply that amount, else the temperature of the body will sink. And our author states, with confidence, the means by which, in his opinion, the calorific effect is produced. Let us, by a severe, but fair, examination, facts being its basis), endeavor to ascertain whether the means referred to, and relied on, by him, are competent to the phenomenon in question?

"Our author alleges, that, in the polar climates, especially during the rigors of winter, man inspires and mingles with his arterial blood, a much larger amount of oxygen, than he does in tropical climates, during the same, or any other, season of the year. And he further alleges, that in the former case, he also swallows, as his food and drink, converts into chyle, and, in like manner, mingles with his *venous*, to be converted into arterial blood, a much larger amount of carbon and hydrogen, than he does in the latter." p. 12.

"Admitting that each hyperborean adult swallows, every day, from ten to fifteen pounds of oily food and drink (against the credibility of which, however, we are compelled to protest), it must be acknowledged, that such a mass of grease conveys into the systems of those who indulge in it, no inconsiderable amount of carbon. Yet do we pronounce that amount far from sufficient to produce the effect essential to the support of our author's hypothesis. It is not, we mean, equal to twenty-eight or thirty times the quantity of the same article that passes into the bodies of the inhabitants of tropical climates. Yet to sustain the notion we are examining, such ought to be the case. The evolution of twenty-eight or thirty times the amount of caloric requires, of course, in the same sort of process, twenty-eight or thirty times the amount of the article from which it is evolved." p. 13.

If the inhabitants of these opposite regions were clothed and lodged alike, there would be force in this argument, but the point of it is destroyed by the fact, that the people of the north compensate, in a great measure, by thick clothing and warm houses, for the absence of the heat of southern latitudes. In northern climates the study of men is to aid the calorific power of their bodies. Every thing is arranged for economising heat. The ice-huts of the Esquimaux were found by Capt. Parry to have a temperature only a few degrees below the freezing point of water, and in these they pass most of their time during the cold season. Instead then of 140° or 150°, the difference between the temperature of the hyperborean's body and that of the medium in which he lives, rarely exceeds and then only for a short time, 68° or 70°; and while he protects himself by furs and the warmest vestments, when exposed to the open air, the inhabitants of warm countries seek a reduced temperature in airy houses, shade, cooling drinks, which favor perspiration, repose, and light clothing. No process cools the body so rapidly as evaporation of the perspirable matter, which flows freely under the influence of a vertical sun, and must be very trifling amid the frosts of a northern winter.

Then, as to the fact that men exposed to cold consume more food, if they can obtain it, and food of a richer quality, it admits of no question. Every one experiences it in his own case. The appetite is proverbially sharp on a frosty morning. We relish a generous animal diet in winter for which we have but little desire in warm weather. Every farmer knows, that his cattle must be better fed, as the cold increases in severity, and that shelters and warm houses compensate for a certain amount of food. "He who is well fed," says Sir John Ross, (*Narrative*, p. 200), resists cold better than the man who is stinted, while the starvation from cold follows but too soon a starvation in food. This, doubtless, explains in a great measure the resisting powers of the natives of these frozen climates; their consumption of food, it is familiar, being enormous, and often incredible." The same traveller asserts, that "an Esquimaux will eat twenty pounds of flesh and oil daily." Captain Cochrane, in his Narrative of a Journey through Russia and Siberian Tartary, states that a calf, weighing about two hundred pounds, "may serve four or five good Yakuti for a single meal," and he declares that he has "repeatedly seen a Yakut or Tongouse devour forty pounds of meat a day." He adds, that he has seen "three of these gluttons consume a reindeer at one meal."

Certainly, here is an ample provision of fuel for the generation of all the caloric required by these people. Whether it be that the "gluttons" are impelled to such feats by a large organ of "*alimentiveness*," or by the instinct of providing fuel for the oxygen they consume, the fact is beyond dispute, that they cannot subsist without "the large use of oil and fat meats, becoming diseased, and dying, with a more meagre diet." (Sir John Ross' *Narrative,* &c.) A diet of such rich articles, observes the same writer, is proved by "all experience to be the true secret of life in these frozen countries."

Dr. Caldwell objects to Liebig, that he does not tell the source whence the natives of the north derive the hydrogen necessary in this process of combustion. The following are his words:

"From what source our author derives his extra-abundance of hydrogen to super-saturate with it the systems of the polar tribes, he does not inform us; and we forbear to inquire. He cannot supply himself with it from the quantity of water those people drink, which we believe to be far inferior to the quantity used by the inhabitants of hot and temperate climates. The latter people, therefore, ought to be much more heated by the combustion of hydrogen in their systems than the former. Yet, the more substantially to fortify his hypothesis, the Professor ought to be prepared to show that the systems of those people, which are so deeply *carbonized*, are also somewhat *hydrogenized*. This condition of them would be the more expedient and useful to him, seeing, as he correctly informs us, the combustion of hydrogen evolves much more caloric, and produces, therefore, a more intense heat, than the combustion of carbon."

This is an unlucky paragraph to occur in a critique on a work relating to chemistry. It was not wise in the author of the Critique to "forbear to inquire," so soon, whether the food of the arctic people might not contain a liberal supply of hydrogen. If he had taken the trouble to consult some elementary treatise on chemistry, he would have learnt that fat animal matters are rich in that substance; or, without going out of the work he was criticising, a little care would have apprised him of Liebig's doctrine, "that it is especially carbon *and hydrogen*, which, by combining with oxygen, serve to produce animal heat." (*Animal Chem.*, p. 23).

Having shown, that the diet of the natives of cold regions abounds in matters fitted for combustion, the next question is, as to the source of oxygen. Dr. Caldwell thus states Liebig's positions in regard to the matter:

"His surplus-supply of oxygen, for the systems of the north-men, our author derives from two several sources, which may be thus stated. 1. A given volume—say a cubic foot—of a cold and dense polar atmosphere, contains more oxygen than an equal volume of a warmer and rarer one, of a temperate or tropical climate. 2. The acts of inspiration in a given time—say a minute—being equally full, are more numerous in a cold climate, and in cold weather, than they are in a temperate or a hot climate, or in temperate or hot weather, in any climate." p. 16.

As respects the first of these positions Dr. C. agrees that Liebig is right; the second he "unhesitatingly pronounces unsound." But on what facts does he rest the assertion? Upon none, except that he "thinks that his pulse-beats in common with his acts of inspiration, are rather less frequent in cold than in hot weather." And perhaps this is true, sitting in his warm parlour. But how stands the case with the people of cold climates, who are urged by their necessities to laborious efforts in the open air? Is it probable, that their "pulse-beats and acts of inspiration" are fewer than those of their brethren at the South? The case is too plain to admit of dispute. The active habits of Northern people, their energy, the inexorable necessity for great exertion in cold climates to supply the demands of nature, are facts universally known. And there cannot, therefore, remain in any mind a question, that both in the frequency of their inspirations, and in the quantity of air inspired each time, the nations of the North receive more oxygen than the inhabitants of Southern countries.

The author of the "Critique" proceeds:

"But other people, besides the fat-meat and fat-fish-eating, and oil-drinking Esquimaux, Samoyedes, and Laplanders, inhabit cold countries and climates, retain in them their temperature, enjoy excellent health, and acquire corresponding degrees of strength and activity. And they do all this with but a meagre supply of animal food, either fat or lean, or of any other sort of oleaginous diet. Nor are their clothing and dwellings by any means of the warmest description." p. 17.

It is manifest from this objection, that its author has not made himself acquainted with Liebig's theory of nutrition and animal heat. The argument rests upon the assumption, that an "oleaginous diet" is indispensable for the supply of the carbon and hydrogen which, according to that theory, are concerned in the combustion of which the heat of the living body is the result. But this is not the fact. "Man," says Liebig, "when confined to animal food, requires for his support and nourishment extensive sources of food,"—five-fold more extensive than when he is furnished with a mixed diet, and for the reason, that "fifteen pounds of flesh contain not more carbon than four pounds of starch." And so the savage, who, with one animal and an equal weight of starch, is now able to maintain life and health for a certain number of days, would be compelled, if confined to flesh, in order to procure the carbon necessary for respiration, during the same time, to consume five such animals. (Liebig, p. 74). What, then, becomes of this objection? It is admitted that the serfs and peasantry of the north of Europe, have "milk and cheese," and bread composed of "rye and oaten meal," and this is all that Liebig's hypothesis demands. In these substances is the most ample supply of hydrogen and carbon. They are the very articles to furnish the elements for combustion; and thus, it turns out, that "the serfs and peasantry of

Russia are," not only not "very scantily," but most abundantly, supplied with Professor Liebig's "*carbonaceous* food."

"We shall here extract," says Professor Caldwell, "from Animal Chemistry, p. 21, another passage, to show the wild and lawless extravagance, in which our author indulges his system-building fancy.

'Our clothing is merely an equivalent for a certain amount of food. The more warmly we are clothed, the less urgent becomes the appetite for food, because the loss of heat by cooling, and consequently the amount of heat to be supplied by food, is diminished.' "

"Of this extraordinary paragraph, the true and literal interpretation is, that we eat, not so much that we may be *nourished* by our aliment, as that we may be *warmed* by it. Consequently, be the temperature of the atmosphere around us what it may—at the freezing point—at zero—or forty degrees below it—provided we swallowed a sufficient amount of food, we may dispense entirely with the use of clothing. In plain English, we may go naked through the most intense degree of cold that can be produced by a frosty atmosphere, and a snow-covered earth, co-operating with the iciest blasts from the pole, and still be sufficiently warm for health and comfort!! Why? Because, if it be true, as Professor Liebig assures us, that 'Our clothing is merely an equivalent for a certain *amount* of food," it follows, of necessity, that that same amount of food is an equivalent for clothing. This conclusion is as certain, as it is that, "things equal to one and the same thing are equal to one another." Only swallow, therefore, a sufficient quantity of bacon, lard, butter, and other sorts of grease, and you may dispense with the cost and the encumbrance of clothes!

"Again, if, according to our assurance from the same authority, it be a fact, that 'the more warmly we are clothed the less urgent becomes our appetite for food;' it follows, of course, that in case our clothing be sufficiently warm, our appetite for food will be entirely extinguished." p. 20.

The best answer, perhaps, that could be given to all this are a few facts resting upon experiment. It is known, for example, that two hives of bees do not consume so much honey when together as when separate, because the warmth is greater. Again, a hundred sheep were placed by Lord Ducie, in a warm shed, and ate twenty pounds of Swedish turnips, each, a day; another hundred kept in the open air consumed, each, twenty-five pounds of turnips daily; yet at the end of a certain period the sheep which had been protected, although they had a fifth less food, weighed three pounds a head more than the unprotected sheep. (Playfair's Lecture before the Royal Agricultural Society of England). From the same paper we derive these additional observations: "During the late riots in Lancashire, the poor unemployed operatives found out that exercise and cold made them hungry; accordingly they kept quiet in bed, and heaped upon them all the covering they could find. Five sheep were fed in the open air between the 21st of November, and the 1st of December; they consumed ninety pounds of food daily, the temperature of the atmosphere being about 44°. At the end of this time they weighed two pounds less than when first exposed. Five other sheep were placed under a shed, and allowed to run about at a temperature of 49°; they consumed at first eighty-two pounds a day; then seventy; and at the end of the time had increased in weight twenty-three pounds."

Could any thing be more conclusive than these experiments? With the supply of warmth from without, the demand for food to generate heat within diminished. But it does not follow, because clothing is an equivalent for a certain amount of food, that, therefore, it might be made to supersede the necessity for food altogether. A man may live upon little, says the proverb, but he cannot live upon nothing at all. A portion of our food, according to Liebig, is consumed in the production of animal heat. Diminish the demand for animal heat, by external warmth, or by clothing, and you diminish, to that extent, the necessity for the kind of food which is concerned in the evolution of animal heat. But you do not, thereby, extinguish the appetite for all food. The author of this objection would have learnt, if he had read Liebig more attentively, that the food of animals consists of two classes; one of which is strictly nutritive, forming blood and supplying it with the elements for the various vital tissues; the other affording the "elements of respiration," or matters to be oxidized, and so, by a slow combustion carried on in every living part, maintain the animal temperature. And he would have seen the injustice of the caricature he has drawn in his attempt to ridicule Liebig upon this point.

The various savage tribes cited by Professor Caldwell, furnish nothing, in their modes of living, contradictory of the theory he opposes. Those of the North subsist upon "venison, fish, Indian corn, and wild rice," from which they derive the necessary "fuel" to sustain their vital heat; and those races inhabiting tropical countries who, nevertheless, are voracious feeders, pursue lives of great activity, thus consuming, or, as expressed by Liebig, "oxidizing" the carbon and hydrogen which, otherwise, would accumulate as fat upon their bodies. Not one fact, in all the cases adduced, but is in perfect harmony with the view, that animal heat results from the union of the elements of the food with the oxygen imbibed in respiration, and that those elements when in quantities disproportioned to the oxygen respired go to form deposits of fat. In the body of the Arab, of the Indian, of the fox, or of the stag, such deposits do not occur, because the food convertible into fat is oxidized by their active respiration—the fuel is burnt up, and that which loads the hog or lazy alderman with fat, escapes from their systems in the form of carbonic acid and water.

The agency of the nervous system in the maintenance of animal temperature, Dr. Caldwell thinks, is not sufficiently recognised by Liebig. He states the case thus:

"Destroy or paralyze the nerves which supply one arm and hand, the circulation of the blood through them continuing, and their temperature will fall several degrees below that of the corresponding limb. Of the lower extremities the same may be said. If the nerves of either of them be seriously deranged, its temperature will decline.

"Paralyze, or seriously injure, the *nervus vagus*, and of all the parts of the body through which it is distributed, the temperature soon and considerably sinks. Nor, in the parts whose nerves are thus deranged, is nutrition carried on to its usual extent; and hence the organs decrease in size. Yet to chemical action does our author ascribe nutrition as well as calorification." p. 24, 25.

Liebig holds calorification to be a chemical process, but one to which nervous influence is necessary. The nerves, once for all, he admits, are essential to all vital actions. Under their influence, the viscera produce the compounds which give rise to animal heat by their union with oxygen. But these compounds are not evolved when the nerves are paralyzed, and the combustion must consequently cease. The nerves being indispensable to the supply of the fuel, it must happen when they are injured that the animal temperature will decline, for the conditions of the chemical action are no longer present. Fuel to be consumed, is as essential as oxygen to consume it, and the cutting of the spinal cord, or of the par vagum, effectually cuts off this supply.

The author of the "Critique" and Liebig are at issue as to the relative temperature of the child and the adult, but here, as usual, the evidence is on the side of the German Professor.

"In infants," says Dr. C., "the lungs are larger, in proportion to the bodies that contain them, than in adults. In the same proportion, therefore, they receive, by each inspiration, a larger quantity of atmospherical air. Their acts of inspiration are also more frequent, and therefore, more numerous in a given time, in the proportion of about 27 or 28 to 19 or 20. Hence, according to our author's hypothesis, the temperature of infants ought to be higher than that of adults. And the gentleman asserts that it is so." p. 26.

Dr. John Davy, (*Researches*, p. 287, 1840), found the temperature of young animals higher than that of animals arrived at maturity. He cites, particularly, observations on infants made by himself. In one instance, he found the heat under the axilla of a child just born 98.5°; after twelve hours 99°, and after three days the same, appearing all the time in perfect health. On five other children of the same age, he made similar observations. In two instances, he says, when the infants were weak, the temperature, one hour after birth, was found not to exceed 96°, which is 2° below the standard of man in health; but their respiration, he adds, was still languid, and the next day the heat of the axilla had risen in one to 98.5°, and in the other to 99°. These observations are conclusive as to this point, nor are they opposed to the results obtained by Edwards in his Researches on Animal Heat, whose inference from all his experiments is, that the small development of heat in the young of carnivorous and rodent animals is due to their small consumption of oxygen. He found, for example, that young sparrows, in which the temperature was lower than in grown sparrows, consumed air much more slowly. And in the young of dogs, cats, and rabbits, which are born blind, and with the respiratory function incomplete, the calorific process was shown to be much less active than in the young Guinea-pig, which can run about and pick up food as soon as it is born. This is in accordance with what is known of the young of the human species. The fœtus, during intra-uterine life, derives its heat from its mother, and the child born prematurely is with difficulty kept warm. In one, respiration has not commenced, in the other is performed inadequately, and in both, as in those children who labor under the *morbus cœruleus*, the facts harmonize perfectly with the chemical theory, that the temperature is in proportion to the extent and activity of the respiratory process. The writings of physiologists abound with evidence to the same point, as the following from Müller: that the larva, in which the respiratory organs are smaller in comparison with the size of the body, has a lower temperature than the perfect insect; that flying insects, which have the largest respiratory organs, have also the highest temperature—and that, among terrestrial insects, those produce the most heat which have the largest respiratory organs and breathe the most air. Nothing more is contended for by Liebig, whose words are, that "the temperature of the child is higher than that of an adult;" and, "a child, in whom the organs of respiration are in a state of great activity, requires food oftener than an adult, and bears hunger less easily"—not, so far as we have been able to find in his Animal Chemistry, that infants are less affected by cold than adults, nor, that the babe may survive the cold by which the mother is frozen to death.

The allusion of the "Critique," in connexion with this subject, to the hybernation of animals, was rather unfortunate, for all the phenomena presented by that state go to support the hypothesis of Liebig. Hybernating animals, when not in a state of torpor, have generally a temperature about as high as that of other animals, but during their sleep the heat of their bodies declines to within four or five degrees of that of the surrounding medium, several of them becoming even frozen at 10° of Fahr. And in this state, what is the condition of their breathing and of their circulation? Respiration is slow, and, at last, almost imperceptible. The marmot during hybernation breaths only seven or eight times in a minute, the hedge-hog four or five times, the great dormouse nine or ten times in the same period. But during the state of the deepest torpor, respiration entirely ceases. *Müller,* p. 77. Sassy found, that the quantity of oxygen consumed, decreased as the temperature of the animals fell; and he found, also, that the motion of the blood in the state of torpor was extremely slow, it being only in the larger vessels that an undulatory motion of this fluid was observable. In the bat, during hybernation, the heart beats but twenty-eight, or, at most but fifty-five times in the minute, while ordinarily it beats two hundred times in the same interval.—*Ib.* p. 78. The temperature of the hedge-hog and dormouse, in their torpid state, was found by Edwards to be 37°, their respiration being scarcely perceptible. When they were roused from their sleep, by mechanical excitement, their breathing became full, and their temperature, in the same cold room, rose in a short time to 86° and 97° Fahr. From all of which, what is the inference? Obviously, none other than that drawn by Edwards, that "increase of respiratory movements and the restoration of heat, stand related as cause and effect." *Edwards on the Influence of Phys. Agents,* &c. p. 97.

The author of the "Critique" is very severe on Liebig's philosophy of starvation. He thinks it is "ludicrous." It may not be amiss to state what that philosophy is.

The first effect of starvation, as every body knows, is the disappearance of the fat of the body, and as the most carefully conducted experiments have shown that it does not pass off by other emunctories, Liebig holds that the hydrogen and carbon of which it is composed are given off through the lungs and skin, in the form of carbonic acid and water. "In the case of a starving man," says he, "32½ oz. of oxygen enter the system daily, and are given out again in combination with a part of his body," and he refers to the case reported by Currie of an individual who was unable to swallow for a month, owing to a schirrous tumor in the œsophagus, and who in that period lost one hundred pounds, and to that of a fat pig overwhelmed in a slip of earth, which lived one hundred and sixty days without food, and was found in that time to have lost one hundred and twenty pounds, to prove that it is the fat which is first oxidized. Dr. Willan reports a similar case. A young man lived fifty-one days upon water with a little orange-juice squeezed into it. Dr. Willan saw him on the sixty-first day of his fast, when his appearance suggested the idea of "a skeleton prepared by drying the muscles upon it in their natural situations." His mind had become imbecile, and he died frantic and exhausted on the seventy-second day from the commencement of his abstinence. He partook of food for several days before his death, but his emaciation continued to increase. In Currie's case the mind was likewise affected, as, indeed, it always is near the approach of death from starvation.

"In the progress of starvation," continues Liebig, "it is not only the fat which disappears, but also, by degrees, all such of the solids as are capable of being dissolved. The muscles are shrunk and unnaturally soft. Towards the end, the particles of the brain begin to undergo the process of oxidation, and delirium, mania and death close the scene." *An. Chem.* p. 25.

This is what our author styles "a ludicrous perversion of the philosophy of starvation," which he attempts to set aside by the following questions:

"On what foundation, and of what materials has Professor Liebig erected his hypothesis? Have their solidity and soundness been thoroughly tested by him? Has he ever analyzed the brain of a person destroyed by famine? If so, has he detected in it a greater amount of oxygen, united to the cerebral matter, than is to be found in the brains of those who had become deranged from any other cause?—or even in the brains of those who had not been deranged at all? If he has not effected such analysis, and made such detection, or found some other authentic evidence in support of his belief (and we are not apprized of his having done either), he is not only unjustifiable, but amenable to censure, for hazarding the assertion." p. 31.

Considering that this critique is avowedly chemical, the passage just quoted is certainly a curious one. Assuredly, the last thing a chemist would expect to find in an oxidized brain, is an excess of oxygen. He knows, that the oxygen has passed away in combination with the carbon and hydrogen of the cerebral matter. Would the author of the "Critique" look for a greater amount of oxygen in the fuel, which is wasting away by oxidation, in his fire-place? He must know, that the process of oxidation, in all such cases, involves the escape of oxygen in a gaseous form. We have ventured upon these brief hints not without misgivings, that we were explaining what was already familiar to every reader.

The objections to this theory of starvation are stated in the following sentences:

"The Professor confidently states, (indeed his hypothesis compels him to state), that, in the bodies of persons destroyed by starvation, the whole of the fat they contain is necessarily burnt out before they die; and that, of course, the greater the quantity they possess of that substance, the longer they live under the torturing privation.

"This is at once a mistake and mis-statement. And it convinces us that either our author is not at all times a correct observer; or that, touching the matter we are now considering, he has had no favorable opportunity to observe. An individual abounding in fat, does not live longer under starvation, than one who does not so abound. Confirmatory of this is the result of observation and experience, as attested by the reports of those, who have witnessed death from starvation, in cases of shipwreck. In such disasters, provided they are equally healthy and vigorous, the lean live as long as the fat, the cheerful and active longer than the dejected and indolent, the firm, resolute, and high-minded, longer than the timid, unresisting, and feeble-minded, and those in the prime of manhood, or still more advanced in life, longer than the youthful—especially than children.

"On this topic we shall further remark, that those who allege (and there are many such), that, under starvation, fat persons outlive lean ones, *because their fatty matter is absorbed and converted into nourishment for them*, are as deeply mistaken in *their* notion, as Professor Liebig is in *his* respecting combustion. We repeat, that fat subjects, when deprived of food and drink, do not live longer than lean ones, for any reason. And we also repeat, that death from starvation is not the result of either *inanition* or *combustion;* but of a *malignant fever*. Nor is it irrelevant to our purpose to subjoin, that, under starvation, those individuals of the inferior animals, that abound in fat, do not live longer than those of the same species that are lean—provided they are alike in all other respects. Experiments to this effect, we have ourselves not only witnessed, but also repeatedly and carefully performed. We therefore speak on the subject with confidence. Nor can aught but a counter-result of experiments, equally well devised and performed, convince us that we are mistaken. We must here remark, however, that the animals experimented on by us, were in no instance actually *destroyed* by starvation. The cruelty of the experiments forbade our pushing them to that extreme. The animals were only so far debilitated as to be *unable to stand. And the fat ones were as much enfeebled as the lean.* On their aliment moreover being restored to them, the latter recruited as rapidly as the former." pp. 32, 33.

"Fat subjects," it is contended, "when deprived of food and *drink*, do not live longer than lean ones;" nor does Liebig say that they do. The time required to cause death by starvation, depends, he says, "on the amount of fat in the body, on the degree of exercise, &c., and on the *presence or absence of water*." We have cited cases in which, water having been used by the sufferer, death did not occur till after the lapse of twenty and sixty days; and the poor Lancashire operatives experienced, that keeping quiet in bed blunted the cravings of hunger. These facts are strikingly coincident with Liebig's "philosophy of starvation," as indeed, are all the facts in our knowledge bearing upon the question, except the experiments reported in the last paragraph above, in which there is much reason to suspect some inaccuracy. Probably the animals were not plentifully supplied with water. But whatever may have been the caution with which they were conducted, we apprehend it will be difficult for the author of them to convince any practical farmer, that his lean pigs will endure starvation as long and as well as the fat ones.

Upon Liebig's hypothesis, it is insisted, that persons with large chests, and who at the same time are full and rich feeders, ought to possess a higher temperature than those of an opposite configuration. No doubt, their power of resisting cold ought to be superior. But, continues the objector,

"Small chested and lunged individuals and sparing eaters of plain and even vegetable food possess a temperature as high as those do, whose chests and lungs are of the largest size; and who eat abundantly of an oily diet." p. 33.

Now, what does experience teach regarding this matter? Our appeal shall be again to practical men—to agriculturists and travellers; and we ask, whether the observation is not universal, that individuals with the largest chests and sharpest appetites, other things being alike, endure fatigue and cold the best? Such, unquestionably, was the observation of Parry and Ross, in their Arctic voyages, and such has been the experience of every man who has had to encounter extreme degrees of cold. We see it every day in man and the inferior animals. A good digestion, and an ample supply of oxygen are the conditions of a high temperature. In syncope, the morbus cœruleus, and dyspepsia, the power to generate heat is low; in the last, because the fuel is insufficient, and in the former two, because there is not an adequate consumption of oxygen.

From patients afflicted with hydrothorax, fevers, pleurisy, and consumption, our author thinks he derives yet stronger testimony against Liebig's hypothesis. In such persons, he remarks, while their respiration is limited, their temperature is not unfrequently increased to febrile heat. But he overlooks the obvious fact, that in these diseases if the breathing is limited it is also much more frequent, and that perspiration being suppressed, evaporation, the great cooling process of the body, is consequently suspended. Müller (*Physiology*, p. 81), admits this to be the cause of the intolerably hot skin in fevers. Carpenter (*Human Physiology*, p. 554), refers the painful heat of the skin, often present in phthisis, to the extremely rapid inspirations which necessarily attend the disorganization of any considerable portion of the lungs. An increased temperature is what ought to occur upon this hypothesis. Apart from the circumstance that evaporation does not take place, the oxidation going on in the body, as shown by the rapid emaciation, ought to develope quite the usual amount of heat. The cooling process being annulled, the temperature might be expected to rise, as it has sometimes been seen in scarlet fever and tetanus, to 106° or 110¾°.

The economy of certain animals is referred to by the author of the "Critique," as subversive of the combustion-theory. Whales, for example, it is said, devour great quantities of food, and yet respire but once in fifteen minutes, but maintain, nevertheless, in northern seas, a temperature of 102°. The anaconda, too, is a voracious feeder, but has a respiration very much restricted. All the serpent tribe, in fact, it is urged, offer this peculiarity.

"Some of that tribe, moreover, if not all of it, possess another peculiarity openly and irreconcilably at war with the notion of Professor Liebig. They are capable of living *months we know*, (and we are assured, on authority we know not how to discredit, or even question, that the term may be extended to *years*), in a state of entire abstinence from food, and of still maintaining their *ordinary temperature.* Nor are they materially reduced in weight by the privation. To invite the disciples of Professor Liebig to reconcile this fact to the combustion-hypothesis of that teacher, might be regarded in the light of an unnecessary taunt. We, therefore, forbear to offer it—leaving them at liberty to attempt or decline the task at their option." p. 35.

We do not doubt, that these examples strike the mind of our author as exceedingly hostile to the hypothesis he is opposing, but we assure him, that they are among the facts upon which Liebig rests his theory. To our minds, indeed, the harmony between this theory and the economy of these animals is perfect. The anaconda devours large meals, it is true, but his rule of feeding, we believe, is the reverse of "*little and often.*" If his meals are hearty, they are far between. After one of his sumptuous repasts, he will go three months without food, and our author thinks "the term might be extended to years." In other words, he comes as near living upon nothing as most animals; and then his lungs are small, and his breathing slow. He is a small consumer both of oxygen and food, and his temperature, as we have seen, in common with that of the serpent tribe in general, is but 2° or 3° higher than that of the surrounding atmosphere. And is not this precisely what the "combustion-hypothesis" requires?

Whales are warm-blooded, and must maintain their independent temperature against a medium which abstracts caloric rapidly. They ought, therefore, to be protected against the cold water by some vestment which conducts heat imperfectly, and be endowed at the same time with organs for developing much animal heat. And so we find their organization. They are large feeders, and although they do not respire often, it does not follow that they are therefore not large consumers of oxygen, the absorption of which, by the lungs, must go on uninterruptedly. The temperature of the animal does not bear any fixed ratio to the number of respirations, in a given time, but to the quantity of oxygen imbibed into the system, and the quantity of carbonic acid exhaled. Thus the horse whose temperature is 98.2° breathes but sixteen times in the minute, while the dog, with a temperature of 99.3°, or one degree higher, breathes twenty-eight times in the same period; man, whose temperature is 99°, breathes eighteen times a minute, but the simia callitriche whose temperature is 95.9° has thirty respirations in the same time; and, more strikingly, the lark breathes but twenty-two times in the minute, while its temperature is 117.2°, being 19° higher than that of the horse, while the number of its respirations is only six more, in the minute. The whale consumes oxygen enough to maintain his vital heat, but not enough to oxidize the whole of his food. Much of that portion of it which is composed of carbon and hydrogen goes to create those deposits of fat, the blubber, which constitute his clothing. If he breathed more oxygen, and consumed all the fuel, such accumulations could not occur, and he would be no longer protected by this admirable inner garment, against the cold of the ice-bergs amid which he passes his life.

The next appeal of the objector is to birds. He remarks,

"As we have long believed, a notion is very generally, if not universally, entertained, respecting the cause of the high temperature of birds, which is not only erroneous in itself, but calculated to infuse error into the whole doctrine of animal heat.

"The temperature of those animals is known to be several degrees higher than that of man, and of quadrupeds. And this superiority in height is attributed to the *supposed* superiority in the extent of their lungs. We say the "supposed superiority;" for we are far from being convinced that it is *real.* On the contrary, we believe that it is not." p. 36.

This is easily answered. The permeability of the bones of birds by the atmosphere being admitted, the chemist asks no more. He knows that the air, once in contact with the moist animal membrane, has no difficulty in reaching the circulation. It does not obviate the difficulty, denying that these air-tubes are part of the true respiratory apparatus. It is enough that oxygen finds its way into them. Even conceding to the objector what all physiologists and anatomists indeed deny, that the respiratory organs of birds are not more extensive than those of the mammalia, the admission avails him nothing, for the truth still remains undisputed and incontrovertible, that more oxygen is consumed, and more carbonic acid generated by birds, in a given time, than by any other class of animals. The quantity formed by cold-blooded animals being as one, by the mammalia it is ten, and by birds nineteen. The mammiferous animals generate fifty times, and birds nearly a hundred times, more than fishes. *Müller*, p. 297–'9. This is all that the supporters of the chemical theory could desire, and thus the conclusion seems to be forced upon the mind, that circumstances so invariably connected as a large, active respiratory apparatus, and a high animal temperature, sustain to each other the relation of cause and effect.

The anaconda and the whale, man, beasts, birds, and fishes having been made to testify against the "combustion-hypothesis," the critique finally calls upon the trees to speak. We are assured that,

"The vegetable kingdom also, abounds in facts in direct opposition to our author's hypothesis. During all the vicissitudes which occur in the atmosphere, the trees of the forest maintain steadily to a certain extent, each kind its own temperature. We mean that they do so as long as they retain their vital condition, but no longer. Thus, during the heat of summer and the cold of winter, the temperature of dead trees accords with that of the atmosphere around them. But not so with trees possessed of life. During warm weather their temperature is considerably *below*, and during cold weather *above*, the temperature of the atmosphere. And of this *vitality* is the cause, independently of any action in the trees of oxygen on either carbon or hydrogen. Nor will the Professor contend that such action exists in them, except perhaps when they are in *positive vegetation*—we mean in actual growth and summer foliage. Assuredly no "combustion" can be even fancied to prevail in them, during the depth of winter, when their roots are surrounded by snow, and their trunks and branches covered with ice. Yet even under the influence of those chilling agents is their temperature retained."

A slight examination of the subject will show, that there is but little truth in this objection. It will be seen, that the difference in temperature between vegetables and the air is generally slight, and not more than may be explained by other causes than the action of "vitality." A fir tree thirteen inches in diameter, on the shores of the Arctic sea, was found by Mr. King to raise the liquid in Fahrenheit's thermometer to 32°, which in the open air stood at 12°, but the earth around the tree at the depth of a foot was 28°, or only 4° below its temperature. This was in October. In May, the temperature of the tree was lower than that of the air, the earth not having yet been warmed as much as the atmosphere. On the 11th of May, the temperature of a fir tree being 34°, that of the air was 40°; on the 12th, another tree showed 33° while the air was at 43°; on the 13th a fir three inches in diameter was at 61°, the atmosphere 55°; the same day, a birch of two and a half inches in diameter and the air agreed in temperature at 55°; on the 16th, a fir four inches in diameter and the air were at the same point, 48°; and on the same day a shrubby birch indicated 63°, while the temperature of the air was 61°. *Captain Back's Narrative*, p. 426, 1836.

Here, it will be remarked, while the temperature of the air was declining, the tree had the advantage in heat, being 4° warmer than the earth about its roots, and many degrees warmer than the surrounding air. Wood is a bad conductor of caloric, and the heat acquired during the previous warm season was given out slowly. The porous bark is a still worse conductor, and this is often aided by a covering of moss, which generally grows thickest on the north sides of trees. These things account for the fact, that at the beginning of cold weather the tree possessed the higher temperature. But as warmth returned in spring the tree was slower than the atmosphere in attaining the mean heat, because the bark and moss kept out the caloric which, a few months before, they had helped to keep in, and it was not until the flow of sap had commenced, and the vegetative process was established, that its temperature exceeded that of the atmosphere. And this process involves chemical action. The conversion of starch into sugar and gum is a chemical process. The temperature does not rise till chemical action begins, and the rise therefore, without violence, may be attributed to chemical action. The truth of this will be rendered nearly certain by what is to follow:

"In the Isle of Bourbon," says Dr. Caldwell, "when the temperature of the atmosphere was but 80° of Fahrenheit, Hubert found the temperature of the flowers of *Arum cordifolium* to be 134°. And it is well known to botanists, that the temperature of the blossoms of sundry plants rises to 119° or 120°—the temperature of the atmosphere at the time being that of summer, in temperate climates. In such cases the blossoms generally grow in clusters.

"How," continues he, "will our author reconcile these phenomena with his hypothesis of vital temperature? Does the combustion of carbon or hydrogen or both take place in these flowers?" p. 38.

Certainly; and we wonder, that a teacher of physiology should ask the question. No fact is better established, than that the flowers of vegetables emit carbonic acid, or, in other words, are the seat of a combustion precisely analogous to that which takes place in the systems of animals. Vegetables, as is well known, grow by absorbing carbonic acid and eliminating oxygen, which is done by their green leaves; but in the process of efflorescence, as in that of germination, the opposite goes on, oxygen is imbibed, and carbonic acid is evolved in great quantities. And, as remarked by Carpenter, (*Physiology*, p. 557), we cannot help being struck by the fact, "that these changes occur with excessive activity at the very periods at which the evolution of heat is most remarkable. "The quantity of oxygen consumed by flowers," continues this writer, "is enormous—those of the Arum Italicum having been found to convert forty times their own bulk of that gas into carbonic acid between the periods of their first appearance and final decay." Is it at all surprising, then, that the temperature of the flowers should be high? Liebig's hypothesis requires that it should; but it is only where these chemical changes are going on, that the high temperature exists. In the leaves and trunks, we have the testimony of most experimenters, that it is but about one degree above that of the surrounding air. It is plain that the author of the "Critique" has pressed this objection through so many pages, from overlooking this obvious fact. Had he considered, as Liebig shows at length, especially in his Vegetable Chemistry, that the function in animals and vegetables, where heat results, is the same—namely the absorption of oxygen, and the extrication of carbonic acid, he would never have published the following paragraph:

"If oxygen can thus, by two modes of action, directly the reverse of each other—union *with* and disunion *from* carbon and hydrogen—produce the same effect, then *may* the hypothesis of Professor Liebig be so far correct. But if it cannot, in its action and influence, thus turn a summerset, then is the hypothesis groundless and untenable. Of this description, therefore, it necessarily is. For as well may it be contended that oxygen, or any other substance, can, at the same time, act and not act, or be and not be, as that it can act to the same effect in two modes, the opposites of one another." p. 40.

We have nothing to say about the experiment with "living and dead wheat." We have seen no report of such an experiment, and until we do, we confess we shall be troubled with doubts, whether a heap of wheat possesses an independent temperature. The fact would be contrary to the observed phenomena throughout both living kingdoms, where, universally, the power of generating heat is associated with a respiratory function. No consumption of oxygen, no independent heat—this seems to be the law in every department of the living world.

But, granting the force of these arguments, what becomes of the "*nervous*" objection? Vegetables have no nerves. If heat is dependent upon nervous action in animals, how is it developed in vegetables? But plants do, in some circumstances, evolve much heat, and this is found to be attendant upon certain chemical operations. Assume, with Liebig, that these operations are "the source of vital heat," and the difficulty disappears.

We come, at length, to "the most herculean objection" to Liebig's theory—the power of the human body to maintain its temperature in a hot atmosphere.

"As far," says Dr. C., "as the reports of experiments inform us, the temperature of the body of man, in a healthy condition, has never been raised above from 100° to 101° or 102° of Fahrenheit. Yet have men, at sundry times, exposed themselves to an atmosphere whose heat ranged from 200° to near 500°.

"About the year 1780, Sir Joseph Banks, Dr. Fordyce, and Dr. Blagden heated three rooms to different degrees, the highest being 260°. To this latter degree, they exposed themselves, both jointly and severally, for a considerable time. Yet did their personal temperature remain stationary at about 100°. Nor was this all.

"Not only did their bodies steadily retain their own temperature; they reduced very materially that of the rooms in which they stood." p. 44.

"Before the time of the performance of these experiments in London, MM. Duhamel and Tilset, two distinguished and enterprising French physicians, exposed themselves, (or rather two young women), with similar effects, to an atmospheric temperature of 325°. And, not many years ago, it was confidently asserted that M. Chaubert (usually called the '*Fire-king*') exposed himself to a heat of about 500°. And still did the temperature of his body remain at 100°.

"We ask our author, or rather his followers and advocates, to reconcile these facts *with* the Professor's hypothesis of animal heat—or to explain them *by* that hypothesis. And we fearlessly assert that they can do neither.

"We know, as we feel persuaded, what *will* and indeed *must* be the reply of Professor Liebig's disciples, provided they venture to give one. They will assert that the abundant exhalation of perspirable matter from the bodies of the experimenters held their temperature within a degree or two of its customary standard. That in two instances it neutralized or rendered latent the influence of 110° and 111° of caloric, above what it manifested itself, in a third 225° and in a fourth 400°. But the assertion is extravagant and wild to the pitch of romance. It is virtually, therefore, a departure not only from truth, but from sober probability. As well may the gentlemen assert that a thimble-full of water is sufficient to quench an eruption of Mount Etna.

"But, as respects the reply of our author's advocates, the worst is to come. Messrs. Banks, Blagden, and Fordyce affirm that, when in the heated rooms, their persons, instead of being *exhaling* bodies, were powerfully *condensing* ones. That they drew from the vapor contained in the atmosphere of the room, and rendered latent in themselves, so much of the caloric which produced it, that the vapor was condensed into water, and, in that form, settled on their bodies, and ran down them in streams." p. 45.

The tone of this is confident enough; nevertheless, we are obliged to say, that the argument has no real force, physiologists themselves being judges. That the facts appear to oppose the chemical theory, is not denied, but it is, we must insist, in appearance only, for when examined it turns out that the effect which strikes one as so wonderful depends, first, upon the air's being a bad conductor of caloric, and, secondly, upon the evaporation of the perspirable matter from the body. Experiment proves it.

For example: The hand may be immersed for a few seconds with impunity in tar at 220°, eight degrees hotter than boiling water. *Annals of Philos.*, vol. ix., p. 3. Suppose it were immersed in boiling water, or in mercury at 220°? The heat of metals is scarcely supportable at 120°—water scalds at 150°. Does the difference depend upon the relative facility with which these bodies convey heat to the body, or upon "a hidden and unknown power" in the human system—"a constitutional instinct"—to "render caloric latent?" This constitutional instinct, for aught that we can see, ought to be as good against the hot water and mercury, as against the melted pitch or heated atmosphere. Why reduce to "latency" the heat in one case, and suffer it to pass with such fatal rapidity in the other? It is a strange "instinct."

The atmosphere is ranked among the worst conductors of heat, an interchange among its particles being the process by which it cools or heats bodies, which process requires time. The bodies of these men were exposed to heat in this medium. The stratum of air in contact with them parted with a portion of its caloric, grew heavier in consequence, and subsided along the floor, making way for a fresh volume of hot air, in its turn to be cooled down and settle towards the bottom. That their temperature rose is evident from the fact, that it was proved to be 100° or 101° after the exposure, and that other animals are found to be heated under a similar exposure; but the increase was not striking, because the evaporation from their surfaces carried away the heat which, if retained, would have soon been insupportable. It is impossible to conceive, that men in health could be exposed to such heat without perspiring most freely, and in every particle of moisture converted into vapor a thousand degrees of heat disappeared. Thus, the caloric slowly imparted by the air, and all excess rendered latent by evaporation, it is not surprising that the experimenters kept cool.

But fancy them in a vapor-bath of 212.° Would their constitutional instinct avail to bring its heat to a state of latency? Delaroche (*Müller*, p. 81), found that if the heated atmosphere be saturated with moisture, which prevents exhalation taking place, the temperature of animals rises 4°, 7°, or 9°, higher than that of the surrounding medium. In other words, their temperature rises as steadily as that of inanimate matter in an atmosphere the humidity of which is sufficient to suppress evaporation, the process by which the redundant heat is carried away, and they soon die in the experiment. In birds, because exhalation is comparatively slight, the heat of the body, according to Delaroche and Berger, rose in a heated air 11° or 12° above the natural standard. These industrious inquirers also found, that an exposure to air of the temperature of 106° to 186°, and especially to hot vapor, speedily raised the heat of their own systems from three to nine degrees. Edwards proved that frogs were killed, in a few minutes, by immersion in water of 104°; and the conclusion is rendered almost certain, that man too would soon perish if heated some 14° above the ordinary temperature of his body. But, in a hot, dry air for the reasons stated, his temperature is not materially raised during the space for which any one has yet remained in it. And thus, we think, the facts brought forward with such an air of triumph, as the "*herculean*" objections to Liebig's theory, are shown to be destitute of any real force. They are, in truth, a fallacy—a sort of optical illusion—and the wonder is, that in the light which modern science has thrown upon them, they should still be urged as arguments against the chemical hypothesis of vital heat.

Chemists cannot be charged with losing sight of the existence of a vital principle in all living beings, which presides over and directs every change in which chemical agency is concerned. Such a principle is distinctly recognised by Liebig. He sets out with declaring its presence, as in the following passage:

"Viewed as an object of scientific research, animal life exhibits itself in a series of phenomena, the connexion and recurrence of which are determined by the changes which the food and the oxygen absorbed from the atmosphere undergo in the organism *under the influence of the vital force. An. Chem.*, p. 9.

But he goes on to add that,

"All vital *activity* arises from the mutual action of the oxygen of the atmosphere and the elements of the food."

This is all intelligible enough. Activity results from the chemical changes, but a principle is resident in vital beings other than a chemical power—"a peculiar force,' as expressed by Liebig, "because it exhibits manifestations which are found in no other force." This force moulds and shapes the organism, directs muscular motion and the secretory processes, and supplies to the oxygen inspired from the air the carbon and hydrogen to be consumed in a slow combustion. This principle extinct, or impaired by an injury of the nerves, the fuel is no longer furnished, and the vital temperature declines.

We have experienced not a little surprise at meeting with the following passage in the "Critique:"

"Can he, (the chemist) at the temperature of 98° or 100° *out* of the animal body, excite combustion in carbon, and form carbonic acid? No, he cannot." p. 91.

We say it surprises us, because when we had the good fortune to be listening to the eloquent lectures of the author of this pamphlet, twenty years ago, we remember to have heard him relate, with great animation, one of his own achievements in chemistry which proves, in the most unequivocal manner, that he did precisely what he now affirms no chemist can do. Professor Woodhouse had denied the possibility of igniting charcoal by nitric acid; but Dr. Caldwell, with a stronger faith, had the happiness of performing the feat, brilliantly, in presence of the Professor, and to his infinite astonishment and delight. Now, during the twenty winters of repeating this anecdote, is it not a little curious, that the Reviewer of Liebig never once suspected, that this might be "a combustion in carbon," with the formation of "carbonic acid," out of the animal body, and at a temperature by many degrees lower? For *cold* nitric acid on *cold* charcoal forms carbonic acid very copiously, and the heat which attends, and finally amounts to a deflagration, is the consequence, not the cause of the chemical action. We will not consume time by referring, in detail, to the germination of seeds, the fermentation of grain, and the putrefaction of animal and vegetable substances, where, as is well known to the youngest chemist, the combination of oxygen and carbon is going on at a temperature, not unfrequently, much below 98° or 100°, and where the extrication of heat accompanies the union. The wonder is, that they should have escaped the attention of a writer who has undertaken to define so exactly the limits of chemical action.

One objection more, and we are done with this part of the "Critique:" The author mentions certain experiments, illustrative of the power of animals to preserve their temperature in hot water, which deserve a passing notice.

"Take," he says, "a large tub-full of water heated to the temperature of 120° of Fahrenheit. Immerse your feet and legs in it, and the sense of burning produced by it will be painful to you. Allow your limbs to be still for a few minutes, and the burning will cease. Remove them to another place in the water several inches distant, and the burning will be reproduced. Hold them again motionless, and again will you be freed from pain." p. 51.

The cause of this is so palpable, that it must have occurred to the mind of the casual reader. It is manifestly due to the circulation of the blood in the limb. The blood being 100° reduces the temperature of the water; and is itself heated at the same time, but as the quantity heated is small in proportion to the mass of that fluid, the general temperature is not sensibly raised in the interval during which the experiment is continued, and might not be increased at all, the perspiratory process and consequent evaporation carrying off the excess of caloric. But the entire body immersed in hot water is found to be heated, even in the few minutes that a hot bath can be endured. Extreme distress follows immersion in such a bath for a short time, and death takes place before the heat of the animal has risen many degrees. *Edwards. Op. Cit. Becquerel and Brechet,* quoted by *Müller.*

The power of gases to penetrate living animal membranes, the author of the "Critique" regards as so questionable, that he "can hardly withhold from it an expression of his disbelief." Liebig's remarks on that subject, he adds, "compel him to suspect the Professor of indiscreet *credulousness*, and of a strong propensity to deal in the *marvellous*." All of which is said in the face of multiplied experiments proving, that animals may be killed by immersing their bodies in poisonous gases, while they are permitted to breathe atmospheric air—that gases pass readily through the bronchi, the diaphragm, and the several coats of the stomach and intestines, the animals alive and in health—experiments as conclusive as any in chemistry, physics, or physiology. And while pronouncing a fact, unquestioned except by himself, to be "wild and extravagant" he hesitates not gravely to put forth the following chimera:

"Admitting it to be true, then, that animals possess a temperature proportioned in height to the extent of their respiration, the fact is to be attributed, not to the superior amount of oxygen, but to that of the *vital principle* received by them in the process." p. 92.

But we have exhausted our limits and must here bring our remarks to a close, incomplete as is our examination of the "Critique." That portion which remains to be noticed appears to us not less objectionable than that which has been passed in review. Truly, the reader of this singular production will have to say, as he turns over its pages,

"———quandoque bonus dormitat Homerus!"

The learned author has slept long upon all the questions to which it relates. Nearly half a century of restless inquiry has swept unheeded by him. Its crowd of discoveries has pressed upon him in vain; his eyes have remained shut to the truths they convey. The theory with which he set out in early professional life continues to be the cherished doctrine of his advanced age, and, like another illustrious theorist in medicine, he seems to have vowed "*never to give it up till he gives up the ghost.*" Y.

